# Effects of supplementation with milk protein on glycemic parameters: a GRADE-assessed systematic review and dose–response meta-analysis

**DOI:** 10.1186/s12937-023-00878-1

**Published:** 2023-10-06

**Authors:** Shooka Mohammadi, Omid Asbaghi, Sina Dolatshahi, Hossein Salehi Omran, Niusha Amirani, Fatemeh Jahangir Koozehkanani, Hossein Bagherzadeh Garmjani, Kian Goudarzi, Damoon Ashtary-Larky

**Affiliations:** 1 Nutrition and Metabolic Diseases Research Center, Ahvaz Jundishapur University of Medical Sciences, Ahvaz, Iran; 2 Department of Social and Preventive Medicine, Faculty of Medicine, University of Malaya, Kuala Lumpur, Malaysia; 3 Cancer Research Center, Shahid Beheshti University of Medical Sciences, Tehran, Iran; 4 Student Research Committee, Shahid Beheshti University of Medical Sciences, Tehran, Iran; 5 Faculty of Medicine, Shahid Beheshti University of Medical Sciences, Tehran, Iran; 6 Faculty of Medicine, Alborz University of Medical Sciences, Tehran, Iran; 7 Department of Clinical Nutrition, School of Nutritional Sciences and Dietetics, Tehran University of Medical Sciences, Tehran, Iran; 8 Department of Nutrition, Maragheh University of Medical Sciences, Maragheh, Iran

**Keywords:** Glycemic indices, High blood glucose (HBG), Hyperglycemia, Milk protein, Whey protein, Casein protein

## Abstract

**Background:**

It is suggested that supplementation with milk protein (MP) has the potential to ameliorate the glycemic profile; however, the exact impact and certainty of the findings have yet to be evaluated. This systematic review and dose–response meta-analysis of randomized controlled trials (RCTs) assessed the impact of MP supplementation on the glycemic parameters in adults.

**Methods:**

A systematic search was carried out among online databases to determine eligible RCTs published up to November 2022. A random-effects model was performed for the meta-analysis.

**Results:**

A total of 36 RCTs with 1851 participants were included in the pooled analysis. It was displayed that supplementation with MP effectively reduced levels of fasting blood glucose (FBG) (weighted mean difference (WMD): -1.83 mg/dL, 95% CI: -3.28, -0.38; *P* = 0.013), fasting insulin (WMD: -1.06 uU/mL, 95% CI: -1.76, -0.36; *P* = 0.003), and homeostasis model assessment of insulin resistance (HOMA-IR) (WMD: -0.27, 95% CI: -0.40, -0.14; *P* < 0.001) while making no remarkable changes in serum hemoglobin A1c (HbA1c) values (WMD: 0.01%, 95% CI: -0.14, 0.16; *P* = 0.891). However, there was a significant decline in serum levels of HbA1c among participants with normal baseline body mass index (BMI) based on sub-group analyses. In addition, HOMA-IR values were significantly lower in the MP supplement-treated group than their untreated counterparts in short- and long-term supplementation (≤ 8 and > 8 weeks) with high or moderate doses (≥ 60 or 30–60 g/d) of MP or whey protein (WP). Serum FBG levels were considerably reduced upon short-term administration of a low daily dose of WP (< 30 g). Furthermore, the levels of serum fasting insulin were remarkably decreased during long-term supplementation with high or moderate daily doses of WP.

**Conclusion:**

The findings of this study suggest that supplementation with MP may improve glycemic control in adults by reducing the values of fasting insulin, FBG, and HOMA-IR. Additional trials with longer durations are required to confirm these findings.

**Supplementary Information:**

The online version contains supplementary material available at 10.1186/s12937-023-00878-1.

## Introduction

The increasing prevalence and burden of type 2 diabetes mellitus (T2DM) and hyperglycemia (very high blood sugar) is a major global health concern [1, 2]. A high blood glucose (HBG) level is a common problem for diabetic patients [2]. Long-term exposure to HBG is the primary causal factor in the pathogenesis of diabetic complications [3]. Hyperglycemia is caused by reduced glucose utilization, increased glucose production, and decreased insulin secretion [4]. It is a potential target to enhance clinical outcomes in hospitalized patients with acute disease, even without overt diabetes [5]. It has been suggested that each 1 mg/ dL increment in fasting glucose concentration may increase the risk of developing diabetes by 9% [6].

Hyperglycemia causes a lot of changes in vascular tissue that could lead to accelerated atherosclerosis [3]. In addition, HBG raises the risk of developing cardiovascular diseases (CVDs) in diabetic and non-diabetic individuals [7]. Furthermore, HBG can be diagnosed in hospitalized patients, even those without diabetes [8]. It can change innate immune responses to infection, leading to poor outcomes in these patients [8]. Therefore, it is essential to monitor blood glucose levels, normalize hyperglycemia, and prevent hyperglycemia-induced complications [9].

Consumption of food products that contain digestible carbohydrates (CHO) can cause postprandial HBG and glycemic responses [10]. A healthy eating pattern is one of the key components of HBG management [11–14]. Cow milk is a food item necessary for a balanced diet and contains several essential micro- and macronutrients [15]. Lactose is the major carbohydrate with a low glycemic index (GI) in dairy products and a disaccharide of glucose and galactose [10]. The lower GI of dairy products has been linked to their matrix for controlling gastric emptying and the presence of lactose [16, 17]. It was reported that the addition of dairy products to high-carbohydrate meals may reduce postprandial blood glucose levels and have a favorable impact on glycemic profile [18]. It decreases post-meal glycemia when consumed during or before an ad libitum meal [19]. In addition, it may deliver comparatively high levels of CHO with limited glycemic responses [10]. However, glycemic reactions following the consumption of milk products are uncertain and controversial [10].

Bovine milk is a major source of high-quality proteins with various nutritional, physiological, and functional benefits [20]. Milk proteins (MP) have health-promoting effects such as digestion and absorption of nutrients, stimulation of the immune system, and prebiotic effects [21, 22]. They may have hypotensive, anticancer, satiating, anti-inflammatory, antimicrobial, antioxidant, and insulinotropic properties [21, 23], as well as the potential to increase muscle protein synthesis [24]. Casein and whey are the main proteins in dairy products that account for 80% and 20% of the amino acids (AAs) in milk, respectively [25]. They are two of the most common types of protein available on the market with different absorption rates and bioavailability [26]. Whey protein (WP) is rapidly digested, whereas casein protein (CP) is classified as a high-quality protein source [23] with slow digestion and absorption [27] that provides all essential AAs to humans, except cysteine [28]. In contrast, WP has a higher proportion of valine, isoleucine, and leucine (essential AAs that are identified as branched-chain amino acids (BCAAs)) than CP [29]. However, non-essential AAs are more abundant in CP [29].

It has been found that proteins are useful in triggering insulin secretion in T2DM patients [30]. The evidence suggests that MP enhances the postprandial insulin response and reduces the postprandial blood glucose response in healthy individuals [31–33] and T2DM patients [1, 34, 35]. The precise mechanisms by which protein of milk lowers the levels of postprandial glucose remain to be determined [36]. The hypothesis is that the AAs and bioactive peptides in MP may lead to delayed gastric emptying, increased incretin and insulin response, and a decrease in postprandial glucose levels [36].

A limited number of reviews and meta-analyses have explored the effects of WP supplementation or dairy products on glycemic control, but they focused only on patients with T2DM [36–39]. The effects of MP supplements on the glycemic profile of different types of consumers were not well investigated and the outcomes of the studies were controversial or inclusive. In addition, meta-analyses of observational studies have higher risks of bias and heterogeneity compared to randomized controlled trials (RCTs) [40]. The main problems in observational studies are confounders and selection bias, but they are prevented in RCTs by blinding and randomization [41]. Therefore, the aim of this systematic review and meta-analysis of RCTs was to assess the impacts of supplementation with MP on glycemic parameters.

## Methods

This systematic review and meta-analysis were performed following the Preferred Reporting Items for Systematic Reviews and Meta-Analyses (PRISMA) framework [42]. The study protocol was registered in the international prospective register of systematic reviews (PROSPERO) (CRD42023424242).

### Search strategy

One reviewer implemented a search strategy to determine relevant RCTs published up to December 2022 in various databases (Medline/ PubMed, Web of Science, and Scopus). The language and period of publications were unrestricted. Google Translate was used to translate articles that were not written in English. The search strategy was focused on four key elements in trials with parallel or crossover design; they were population (adult), exposure/intervention (MP supplement), comparator/control (no intervention or placebo), and outcomes (levels of fasting blood glucose (FBG), hemoglobin A1c (HbA1c), homeostasis model assessment of insulin resistance (HOMA-IR), and fasting insulin). The subsequent medical subject headings (MeSH) and non-MESH were used in the search strategy: (("milk" OR "milk protein" OR "milk protein supplement" OR "milk protein supplementation" OR "whey" OR "casein" OR "whey supplement" OR "whey supplementation" OR "casein supplement" OR "casein supplementation" OR "milk protein concentration" OR "MPC") AND ("glucose tolerance" OR "insulin resistance" OR "FBG" OR "fasting blood glucose" OR "HbA1c" OR "hemoglobin A1c" OR "HOMA-IR" OR "homeostatic model assessment" OR "Insulin" OR "fasting blood sugar" OR "FBS") AND ("Intervention" OR "Intervention Study" OR "Intervention Studies" OR "controlled trial" OR "randomized" OR "randomised" OR "random" OR "randomly" OR "placebo" OR "clinical trial" OR "Trial" OR "randomized controlled trial" OR "randomized clinical trial" OR "RCT" OR "blinded" OR "double-blind" OR "double blinded" OR "trial" OR "clinical trial" OR "trials" OR "Pragmatic Clinical Trial" OR "Cross-Over Studies" OR "Cross-Over" OR "Cross-Over Study" OR "parallel" OR "parallel study" OR "parallel trial")).

### Study selection criteria

Identified records were exported to the Endnote reference management software. Two reviewers (SM and SD) independently assessed the studies and determined appropriate RCTs based on the inclusion criteria. They discussed any disagreements or resolved them through negotiation with a third investigator (DAL). This systematic review and meta-analysis included all RCTs (with crossover or parallel design) that looked at the effect of MP administration on serum levels of HbA1c, fasting insulin, HOMA-IR, and FBG in MP supplement-treated individuals compared with their untreated counterparts.

Eligible RCTs enrolled adult individuals and had a cross-over or parallel design, as well as a placebo or control group. They had a pre-post design with a duration longer than two weeks. In addition, the RCTs had sufficient data on the values of HOMA-IR, fasting insulin, HbA1c, and FBG in the MP-treated and placebo groups at the end of each study and baseline. The trials evaluated the impact of supplementation with MP on the glycemic parameters in participants. Furthermore, the MP supplement was not administered as a multi-component supplement in the MP-treated and placebo groups. Moreover, RCTs with one of the following criteria were excluded: non-placebo-controlled or uncontrolled trials; studies that included individuals under 18 years of age or pregnant women; RCTs with < 2 weeks in duration; non-RCTs or observational studies; trials with inadequate data on selected outcomes at follow-up or baseline assessments.

### Data extraction

Two independent researchers (SM and HSO) extracted data from eligible full-text articles to determine the required information; disagreements were resolved through discussion. The extracted data were related to study characteristics (sample size, publication year, trial duration and setting, study design, dose of MP supplement, type of placebo or control group, and first author’s name), and participants’ demographics (mean body mass index (BMI), age, and gender). In addition, pre- and post-assessments of selected outcomes (HOMA-IR, FBG, HbA1c, and fasting insulin) were collected at the endpoints and baseline of the study.

### Risk of bias assessment

Two independent researchers (SM and NA) appraised the quality of the trials based on the modified Cochrane risk of bias (RoB 2) tool [43]. It identified possible causes of bias including attrition bias, performance bias, allocation bias, reporting bias, and detection bias. The RoB for each domain was deemed high, unclear, and low [43].

### Certainty assessment

The certainty of the evidence was assessed by applying the GRADE (Grading of Recommendations Assessment, Development, and Evaluation) approach, which categorizes the quality of evidence as moderate, very low, low, and high [44].

### Statistical analysis

Meta-analysis was performed by applying the STATA statistical software (version 17). The effects of MP administration on the glycemic parameters were measured as a 95% confidence interval (CI) and weighted mean differences (WMDs) for total changes of trial outcomes from baseline to endpoints in the MP-treated and untreated groups. The outcome measures were presented as standard deviation (SD) and mean. The effect sizes were determined by the mean differences. The following formula was applied to calculate SD changes from pre-to post-intervention: SD change = √ (SD^2^_baseline_ + SD^2^_final_)– (2 × R _correlation coefficient_ × SD _baseline_ × SD _final_) [45]. The random-effects model was employed to calculate the pooled WMDs [46]. The heterogeneity among RCTs was evaluated by applying the I^2^ statistic [47] and Cochrane’s Q test. The I^2^ values 25–50%, < 25%, 50–75%, and > 75%, were considered as moderate, low, high, and very high heterogeneity between RCTs, respectively [48].

Sub-group analyses were applied to identify the possible sources of heterogeneity among the included RCTs. The analysis was based on baseline serum levels of the outcomes (HbA1c, fasting insulin, HOMA-IR, and FBG), trial duration (> 8 weeks vs. ≤ 8 weeks), the dose of MP supplement (≥ 60 g/d vs. 30–60 g/d vs. < 30 g/d), protein supplementation type (WP vs. CP vs. MP), gender (male vs. both female and male vs. female), and baseline BMI of participants (overweight (25–29.9 kg/m^2^) vs. obese(> 30 kg/m^2^) vs. normal (18.5–24.9 kg/m^2^)). Leave-one-out sensitivity analyses were utilized to determine the effect of each study on the overall analysis. In addition, funnel plots, Egger's [49], and Begg's tests [50] were employed to determine probable publication bias. In addition, a *P*-value less than 0.05 was reported as statistically significant. The fractional polynomial model was used to find the possible non-linear impacts of the dose of MP supplement (g/d) and the trial duration (weeks). In addition, meta-regression was performed to evaluate a dose–response slope for a potential linear relationship between effect sizes, trial length, and dose of MP supplement [51].

## Results

### Study selection

A primary search among multi-databases yielded 15,632 records. After excluding 5238 duplicate studies, 10,394 records were screened, and 10,287 citations were excluded based on their titles and abstracts. Full texts of 107 articles were assessed, and 36 eligible RCTs that met the inclusion criteria were analyzed in this study. A flowchart of the study selection and screening process is illustrated in Supplemental Fig. 1.

### Study characteristics

The present systematic review and meta-analysis included 36 trials. Characteristics of the included RCTs are presented in Table 1. Thirty-four RCTs had parallel designs [52–85], while two were cross-over trials [86, 87]. The total number of participants in all trials was 1851 (MP supplement-treated group, n = 975; controls, n = 992), with mean age and BMI ranging from 18 to 85 years and 20 to 37 kg/m^2^, respectively. The sample sizes ranged from 16 to 171 participants. Twenty RCTs [52–58, 62, 63, 66, 70, 71, 74–76, 79, 80, 82, 83, 87] used a mixed-sex sample, while seven and nine studies had a women-only sample [64, 68, 69, 77, 78, 81, 86] or a men-only sample [59–61, 65, 67, 72, 73, 84, 85], respectively.

**Table 1:** Characteristics of included studies in the meta-analysis

**Author, year**	**Country**	**Study Design**	**Participants**	**Sex**	**Sample size**		**Trial Duration (Weeks)**	**Means Age**		**Means BMI**				
					**IG**	**CG**		**IG**	**CG**	**IG**	**CG**	**Type**	**Supplement dose (g/day)**	**CG**
Lee et al. 2007 [52]	Germany	Parallel, R, PC, DB	Patients with mild HTN	♂/♀	25	25	12	55.3 ± 10.4	47.8 ± 11.6	28.5 ± 4.2	27.2 ± 4	Milk	3.38	Skim milk without whey
Keogh & Clifton. 2008 [53]	Australia	Parallel, R, PC, DB	Individuals with overweight and obesity	♂/♀	34	38	48	49.6 ± 12.3	50.3 ± 12.4	34.4 ± 3.7	34.4 ± 3.7	Whey	15	Skim milk powder
Claessens et al. 2009 (a) [54]	Netherlands	Parallel, R, PC	Individuals with overweight and obesity	♂/♀	14	16	12	45.4 ± 8.2	46 ± 8.8	32.9 ± 6	32.4 ± 4.8	Casein	50	Maltodextrin
Claessens et al. 2009 (b) [54]	Netherlands	Parallel, R, PC	Individuals with overweight and obesity	♂/♀	18	16	12	44.9 ± 8.5	46 ± 8.8	33.4 ± 4	32.4 ± 4.8	Whey	50	Maltodextrin
Silva et al. 2010 [55]	Brazil	Parallel, R, CO, DB	Patients with ALS	♂/♀	8	8	16	53	53	21.7 ± 1.1	22.9 ± 1.1	Whey	22	Maltodextrin
Pal et al. 2010 (a) [86]	Australia	Crossover, R, CO, SB	Post-menopausal women with overweight	♀	20	20	3	< 66	< 66	25–40	25–40	Whey	45	Glucose
Pal et al. 2010 (b) [86]	Australia	Crossover, R, CO, SB	Post-menopausal women with overweight	♀	20	20	3	< 66	< 66	25–40	25–40	Casein	45	Glucose
Pal et al. 2010 (a) [56]	Australia	Parallel, R, CO, SB	Individuals with overweight and obesity	♂/♀	25	25	12	18–65	18–65	32 ± 4	30.6 ± 4.5	Whey	54	Glucose
Pal et al. 2010 (b) [56]	Australia	Parallel, R, CO, SB	Individuals with overweight and obesity	♂/♀	20	25	12	18–65	18–65	31.3 ± 4.5	30.6 ± 4.5	Casein	54	Glucose
Takahira et al. 2011 [57]	Japan	Parallel, R, PC, DB	Individuals with visceral fat obesity	♂/♀	23	21	32	54.4 ± 13	56.8 ± 12.2	29.3 ± 3.8	29 ± 4.5	Milk	9	Soy protein
Gouni-Berthold et al. 2012 [58]	Germany	Parallel, R, PC, DB	Patients with metabolic syndrome	♂/♀	83	88	12	52.9 ± 10.3	53.9 ± 9.5	30.8 ± 4.2	31.3 ± 4	Whey	15	Yogurts
Hambre et al. 2012 [59]	Sweden	Parallel, R, CO	Healthy males	♂	12	12	12	24.2 ± 3.7	23.2 ± 3.4	22.6 ± 2.5	22.3 ± 1.9	Whey	33	A meal of fast food
Sheikholeslami Vatani et al. 2012 [60]	Iran	Parallel, R, PC, SB	Young men with overweight	♂	9	10	6	23 ± 2	21 ± 1	26.5 ± 1.2	27.2 ± 1.6	Whey	90	Starch
Ahmadi Kani Golzar et al. 2012 [61]	Iran	Parallel, R, PC, SB	Young men with overweight	♂	10	10	6	22.7 ± 2.3	21.20 ± 1.03	26.5 ± 1.1	27.1 ± 1.5	Whey	30	Starch solution
Björkman et al. 2012 [62]	Finland	Parallel, R, CO	Nursing home residents	♂/♀	46	51	24	84.1 ± 7.6	83 ± 8.7	24.8 ± 4.3	24 ± 5.5	Whey	20	Regular juice
Rambousková et al. 2014 [63]	Czech Republic	Parallel, R, CO	Elderly individuals	♂/♀	23	24	8	84.2 ± 9.7	85.3 ± 9.2	20.3 ± 2.9	20.4 ± 2.8	Milk	18.2	Non-intervention
Piccolo et al. 2015 [64]	USA	Parallel, R, CO, DB	Women with obesity	♀	16	11	8	41 ± 9.8	41 ± 9.8	36.9 ± 3.1	36 ± 4.8	Whey	20	Gelatin
Tahavorgar et al. 2015 [65]	Iran	Parallel, R,CO, DB	Men with overweight and obesity	♂	26	19	12	39.4 ± 6.9	38.8 ± 8.8	32.1 ± 3.2	32.1 ± 2.7	Whey	65	Soy protein
Fekete et al. 2016 (a) [87]	UK	Crossover, R, CO, DB	Healthy individuals with mildly elevated BP	♂/♀	38	38	8	52.9 ± 12.9	52.9 ± 12.9	27.1 ± 4.9	27.1 ± 4.9	Whey	56	Maltodextrin
Fekete et al. 2016 (b) [87]	UK	Crossover, R, CO, DB	Healthy individuals with mildly elevated BP	♂/♀	38	38	8	52.9 ± 12.9	52.9 ± 12.9	27.1 ± 4.9	27.1 ± 4.9	Casein	56	Maltodextrin
Arciero et al. 2016 [66]	USA	Parallel, R, CO	Individuals with overweight	♂/♀	12	9	16	48 ± 12	52 ± 4	32 ± 7	33 ± 3	Whey	25	Food protein
Maltais et al. 2016 [67]	Canada	Parallel, R,CO, DB	Sarcopenic elderly men	♂	8	8	16	68 ± 5.6	64 ± 4.8	25.8 ± 3	27 ± 2.7	Milk	13.53	Soy milk
Stojkovic et al. 2017 [68]	USA	Parallel, R, PC, DB	Postmenopausal women	♀	38	46	72	68.9 ± 5.5	69.3 ± 6.1	26 ± 3.7	25.8 ± 4.1	Whey	20	Maltodextrin
Lopes Gomes et al. 2017 [69]	Brazil	Parallel, R, CO	Patients after bariatric surgery (> 24 months)	♀	15	15	16	41 ± 10	49 ± 10	36 ± 6	35 ± 4	Whey	46	Non-intervention
Hassan et al. 2017 [70]	Israel	Parallel, R, CO	Hypoalbuminemia peritoneal dialysis patients	♂/♀	18	18	12	59.7 ± 11.5	58.1 ± 12.3	28.7 ± 3.3	28.6 ± 3.5	Whey	26.3	Protein without whey
Ottestad et al. 2017 [71]	Norway	Parallel, R, PC, DB	Older adults	♂/♀	17	19	12	76.8 ± 6.2	77.1 ± 4.7	27.6 ± 4.2	25.9 ± 4.9	Milk	40	Carbohydrate
Lockwood et al. 2017 (a) [72]	USA	Parallel, R, PC, DB	Healthy college-aged males	♂	15	15	8	21.8 ± 3.5	20.9 ± 1.5	NR	NR	High-lactoferrin-Whey	60	Carbohydrate
Lockwood et al. 2017 (b) [72]	USA	Parallel, R, PC, DB	Healthy college-aged males	♂	13	15	8	21.3 ± 2.5	20.9 ± 1.5	NR	NR	Whey	60	Carbohydrate
Lockwood et al. 2017 (c) [72]	USA	Parallel, R, PC, DB	Healthy college-aged males	♂	13	15	8	21.5 ± 3.2	20.9 ± 1.5	NR	NR	Extensively hydrolyzed whey	60	Carbohydrate
Gaffney et al. 2018 [73]	New Zealand	Parallel, R, PC, DB	Patients with T2DM	♂	12	12	10	53.5 ± 5.6	57.8 ± 5.2	29.6 ± 2.7	30.1 ± 4.9	Whey	28.5	Carbohydrate
Sharp et al. 2018 [74]	USA	Parallel, R, CO, DB	Healthy participants	♂/♀	10	10	8	19 ± 2	21 ± 2	NR	NR	Whey	46	Maltodextrin
Larsen et al. 2018 [75]	Denmark	Parallel, R, CO, SB	Individuals with overweight and obesity	♂/♀	14	15	4	41	41	34.9 ± 5.4	35.1 ± 5.8	Whey	41	Maltodextrin
Derosa et al. 2019 [76]	Italy	Parallel, R, PC, DB	Patients with T2DM	♂/♀	59	58	12	59.7 ± 9.1	58.6 ± 8.8	22.7 ± 2.1	22.7 ± 2.1	Whey	9.25	Placebo (5 g caseins)
Nabuco et al. 2019 [77]	Brazil	Parallel, R, PC, DB	Older women with sarcopenic obesity	♀	13	13	12	68 ± 4.2	70.1 ± 3.9	26.4 ± 3	27.4 ± 3	Whey	35	Maltodextrin
Giglio et al. 2019 [78]	Brazil	Parallel, R, PC, DB	Women with overweight	♀	17	20	8	37.8 ± 12	43 ± 8	31.1 ± 4	30.9 ± 3.6	Whey	40	Collagen
Yang et al. 2019 (a) [79]	China	Parallel, R, CO	Individuals with pre or mild HTN, and normal weight	♂/♀	12	12	12	42.37 ± 11.6	43.8 ± 11.7	24.1 ± 3.1	24.3 ± 2.3	Whey	30	Maltodextrin
Yang et al. 2019 (b) [79]	China	Parallel, R, CO	Individuals with pre or mild HTN, overweight, and obesity	♂/♀	15	15	12	42.37 ± 11.6	43.8 ± 11.7	24.1 ± 3.1	24.3 ± 2.3	Whey	30	Maltodextrin
Hudson et al. 2020 [80]	USA	Parallel, R, PC, DB	Individuals with overweight and obesity	♂/♀	21	23	16	53 ± 9.2	52 ± 4.8	31 ± 3.2	30.3 ± 3.4	Milk	64	Maltodextrin
Haidari et al. 2020 [81]	Iran	Parallel, R, CO	Pre-menopausal women with obesity on a weight-loss diet	♀	30	30	8	31 ± 6.2	32.2 ± 5.1	33.5 ± 3.17	33.3 ± 2.6	Whey	30	Isocaloric weight-loss diet
Lefferts et al. 2020 [82]	USA	Parallel, R, CO, DB	Older adults	♂/♀	53	46	12	69 ± 7	67 ± 6	27.9 ± 5.6	27 ± 3.9	Whey	50	Carbohydrate
Fuglsang-Nielsen et al. 2021 (a) [83]	Denmark	Parallel, R, CO, DB	Individuals with abdominal obesity	♂/♀	15	16	12	64	64	NR	NR	Whey + low fiber	60	Maltodextrin + low fiber
Fuglsang-Nielsen et al. 2021 (b) [83]	Denmark	Parallel, R, CO, DB	Individuals with abdominal obesity	♂/♀	17	17	12	64	64	NR	NR	Whey + high fiber	60	Maltodextrin + high fiber
Pettersson et al. 2021 [84]	Sweden	Parallel, R, PC.DB	Untrained men with overweight or obesity	♂	10	10	6	28.2 ± 5.5	27.9 ± 5	29.8 ± 2.3	30.4 ± 1.8	Milk	10	Carbohydrate
Teixeira et al. 2022 [85]	Portugal	Parallel, R, CO, DB	Futsal players	♂	20	20	8	18–35	18–35	NR	NR	Whey	25	Plant-based protein

*Abbreviations: IG* Intervention group, *CG* Control group, *DB* Double-blinded, *SB* Single-blinded, *PC* Placebo-controlled, *CO* Controlled, *R* Randomized, *NR* Not reported, ♀ Female, ♂ Male, *ALS* Amyotrophic lateral sclerosis, *BP* Blood pressure, *T2DM* Type 2 diabetes mellitus, *HTN* Hypertension, *BMI* Body mass index*, USA* the United States of America, *UK* the United Kingdom

The trials enrolled patients with pre-or mild hypertension [52, 79], amyotrophic lateral sclerosis (ALS) [55], metabolic syndrome [58], after bariatric surgery (> 24 months) [69], hypoalbuminemia on peritoneal dialysis [70], T2DM [73, 76], and sarcopenic elderly men [67]. In addition, the RCTs were carried out among participants with overweight or obesity [53, 54, 56, 60, 61, 64–66, 75, 78, 80, 84], visceral fat [57], or abdominal obesity [83], and post-menopausal women [68] with overweight [86] or obesity [81]. The studies also included older women with sarcopenic obesity [77], futsal players [85], nursing home residents [62], elderly adults [63, 71, 82], and healthy individuals [59, 72, 74] with mildly elevated blood pressure (BP) [36].

The articles were published between 2007 and 2022. The RCTs were performed in Germany [52, 58], Australia [53, 56, 86], Netherlands [54], Brazil [55, 69, 77, 78], Japan [57], Portugal [85], Sweden [59, 84], and Iran [60, 61, 65, 81]. The settings of studies were also Finland [62], the Czech Republic [63], the United States(US) [64, 66, 68, 72, 74, 80, 82], the United Kingdom(UK) [87], Canada [67], Israel [70], Norway [71], New Zealand [73], Denmark [75, 83], Italy [76], and China [79]. The length of the trials was between 3 and 72 weeks and the doses of MP, WP, or CP supplements ranged from 3.5 to 90 g per day. The risk of bias evaluation among 36 RCTs is displayed in Supplemental Table 1.

The GRADE evaluation of the overall certainty of the evidence for the measured outcomes is summarized in Supplemental Table 2. The HbA1c outcome was downgraded to low quality due to serious limitations in inconsistency and imprecision. High certainty of evidence was allocated to fasting insulin outcome. In addition, moderate quality evidence was considered for FBG and HOMA-IR outcomes because of a very serious risk of inconsistency or serious limitations in publication bias and inconsistency, respectively.

**Table 2:** Subgroup analyses of supplementation with milk protein on glycemic parameters

Sub-groups	Effect size, n	WMD (95%CI)^a^	P-within subgroups			
				**P-heterogeneity**^**c**^	**I**^**2**^** (%)**^**b**^	**P-between subgroups**
Supplementation with milk protein on FBG (mg/dL)						
Overall effect	42	-1.83 (-3.28, -0.38)	**0.013**	< 0.001	80.3%	
FBG Baseline						
< 100	28	-1.25 (-2.86, 0.35)	0.127	< 0.001	79.1%	0.110
> 100	12	-4.48 (-8.09, -0.87)	**0.015**	< 0.001	84.9%	
Trial duration (week)						
> 8	25	-1.49 (-3.18, 0.20)	0.084	< 0.001	76.6%	0.438
≤ 8	17	-2.74 (-5.43, -0.06)	**0.045**	< 0.001	84.4%	
Intervention type						
Casein	4	-7.53 (-16.60, 1.54)	0.104	< 0.001	92.6%	0.267
Milk	9	-0.82 (-2.49, 0.84)	0.333	0.149	33.7%	
Whey	29	-2.00 (-3.77, -0.23)	**0.026**	< 0.001	81.1%	
Supplement dose (g/day)						
≥ 60	5	-0.19 (-2.65, 2.25)	0.876	0.265	23.5%	0.298
30–60	24	-1.62 (-3.63, 0.39)	0.114	< 0.001	81.1%	
< 30	10	-3.21 (-6.12, -0.29)	**0.031**	< 0.001	81.0%	
Baseline BMI (kg/m^2^)						
Normal (18.5–24.9)	5	-6.00 (-9.48, -2.53)	**0.001**	0.005	73.1%	0.010
Overweight (25–29.9)	14	-4.87 (-8.41, -1.33)	**0.007**	< 0.001	84.1%	
Obese (> 30)	14	-0.28 (-2.55, 1.97)	0.803	< 0.001	79.9%	
Sex						
Both	24	-0.90 (-2.61, 0.80)	0.299	< 0.001	76.4%	0.013
Female	7	-10.87 (-17.28, -4.45)	**0.001**	< 0.001	92.4%	
Male	11	-1.32 (-3.00, 0.36)	0.125	0.080	40.3%	
Supplementation with milk protein on fasting insulin (uU/ml)						
Overall effect	24	-1.06 (-1.76, -0.36)	**0.003**	0.003	50.1%	
Trial duration (week)						
> 8	15	-0.93 (-1.70, -0.17)	**0.017**	0.046	41.7%	0.669
≤ 8	9	-1.31 (-2.87, 0.24)	0.098	0.006	63.0%	
Intervention type						
Casein	3	-2.85 (-8.80, 3.09)	0.347	0.024	73.2%	0.797
Milk	5	-1.38 (-3.30, 0.54)	0.160	0.157	39.6%	
Whey	16	-1.02 (-1.79, -0.24)	**0.010**	0.010	50.9%	
Supplement dose (g/day)						
≥ 60	4	-1.71 (-2.68, -0.75)	** < 0.001**	0.691	0.0%	0.019
30–60	11	-1.59 (-2.78, -0.41)	**0.008**	0.014	55.0%	
< 30	8	0.01 (-0.89, 0.92)	0.975	0.236	24.2%	
Baseline BMI (kg/m^2^)						
Normal	3	-0.56 (-2.46, 1.33)	0.559	0.013	76.9%	0.847
Overweight (25–29.9)	9	-0.91 (-2.33, 0.50)	0.207	0.040	50.5%	
Obese (> 30)	10	-1.23 (-2.54, 0.08)	0.066	0.054	46.1%	
Sex						
Both	15	-0.74 (-1.39, -0.09)	**0.025**	0.140	28.9%	0.742
Female	6	-1.66 (-4.10, 0.78)	0.182	0.014	64.9%	
Male	6	-1.23 (-3.87, 1.41)	0.362	0.035	70.2%	
Overall effect	6	0.01 (-0.14, 0.16)	0.891	< 0.001	82.2%	
HbA1c Baseline						
< 6.4	4	0.06 (-0.01, 0.13)	0.123	0.429	0.0%	0.302
> 6.4	2	-0.13 (-0.48, 0.22)	0.471	0.003	88.8%	
Intervention type						
Casein	1	0.18 (-0.01, 0.37)	0.075	-	-	0.247
Milk	1	0.10 (-0.08, 0.28)	0.295	-	-	
Whey	4	-0.04 (-0.23, 0.13)	0.619	< 0.001	85.1%	
Supplement dose (g/day)						
30–60	2	0.12 (-0.00, 0.25)	0.062	0.451	0.0%	0.159
< 30	4	-0.04 (-0.23, 0.14)	0.655	< 0.001	85.1%	
Baseline BMI (kg/m^2^)						
Normal	1	-0.30 (-0.42, -0.17)	** < 0.001**	-	-	< 0.001
Overweight (25–29.9)	2	0.08 (-0.05, 0.21)	0.245	0.776	0.0%	
Obese (> 30)	3	0.06 (-0.03, 0.15)	0.231	0.279	21.8%	
Supplementation with milk protein on HOMA-IR						
Overall effect	20	-0.27 (-0.40, -0.14)	** < 0.001**	0.006	49.9%	
HOMA-IR Baseline						
> 2	11	-0.29 (-0.49, -0.09)	**0.005**	0.150	31.2%	0.815
< 2	8	-0.26 (-0.46, -0.05)	**0.014**	0.002	69.0%	
Trial duration (week)						
> 8	14	-0.25 (-0.41, -0.10)	**0.001**	0.015	50.9%	0.752
≤ 8	6	-0.31 (-0.58, -0.03)	**0.027**	0.085	48.2%	
Intervention type						
Casein	2	-0.20 (-0.85, 0.44)	0.540	0.196	40.1%	0.383
Milk	3	-0.53 (-0.89, -0.16)	**0.004**	0.650	0.0%	
Whey	15	-0.25 (-0.40, -0.11)	**0.001**	0.005	55.2%	
Supplement dose (g/day)						
≥ 60	4	-0.41 (-0.60, -0.22)	** < 0.001**	0.732	0.0%	0.017
30–60	8	-0.36 (-0.54, -0.18)	** < 0.001**	0.331	12.6%	
< 30	7	-0.08 (-0.24, 0.08)	0.347	0.126	39.8%	
Baseline BMI (kg/m^2^)						
Normal (18.5–24.9)	2	-0.42 (0.67, -0.17)	**0.001**	0.333	0.0%	0.369
Overweight (25–29.9)	7	-0.17 (-0.41, 0.06)	0.147	0.088	45.5%	
Obese (> 30)	9	-0.27 (-0.47, -0.06)	**0.010**	0.142	34.5%	
Sex						
Both	11	-0.31 (-0.45, -0.18)	** < 0.001**	0.631	0.0%	0.925
Female	5	-0.25 (-0.56, 0.05)	0.106	0.061	55.6%	
Male	4	-0.32 (-0.79, 0.13)	0.165	0.002	79.1%	

*Abbreviations:* *WMD* weighted mean differences, *CI* confidence interval, *BMI* body mass index, *HOMA-IR* Homeostatic Model Assessment for Insulin Resistance, *FBG* fasting blood glucose, *HbA1c* hemoglobin A1c, *CI* Confidence interval

^a^Attained from the random-effects model

^b^Percentage of differences between trials due to heterogeneity

^c^Cochrane’s Q test

### Effect of supplementation with milk protein on serum FBG

**Fig. 1:**
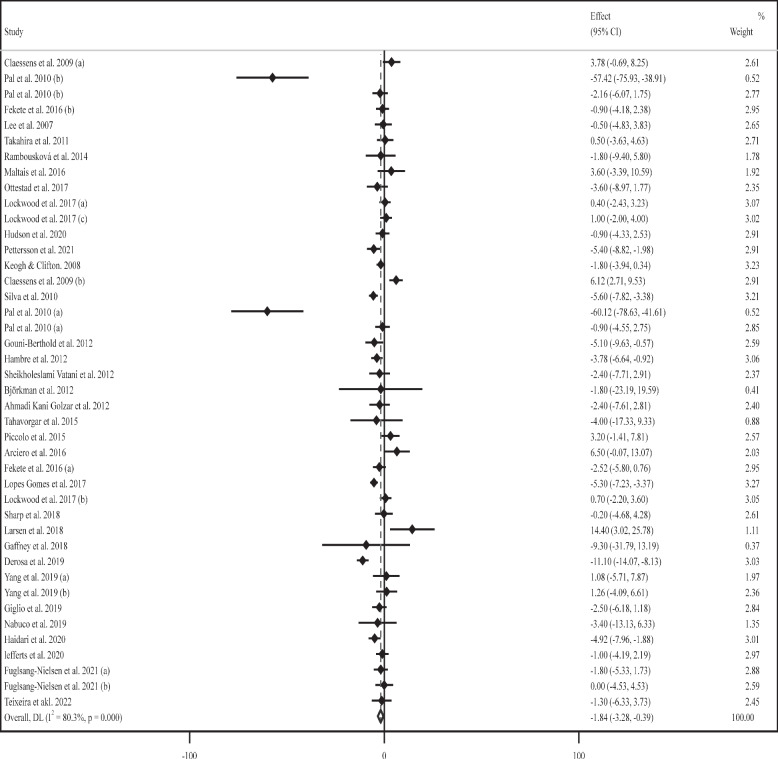
Forest plot for the effect of supplementation with milk protein on fasting blood glucose (FBG) (mg/dL). Horizontal lines represent 95% confidence intervals (CIs). Diamonds represent pooled estimates from random-effects analysis. The effect column comprises weighted mean differences (WMDs) and 95% CIs

Thirty-four RCTs (42 trial arms) [52–67, 69, 71–87] with 1731 participants (MP-treated group, n = 919; placebo group, n = 928) were included in this meta-analysis. The pooled analysis displayed that MP supplementation effectively reduced serum concentrations of FBG in the MP supplement-treated group compared with their untreated counterparts (WMD: -1.83 mg/dL, 95% CI: -3.28, -0.38; *P* = 0.01). In addition, there was considerable heterogeneity between trials (*I*^*2*^ = 88.3%, *P* < 0.001) (Fig. 1). Sub-group analyses explored that serum FBG levels were considerably reduced upon short-term administration (≤ 8 weeks) of a low daily dose of WP (< 30 g) among female participants with normal or overweight BMI and higher baseline FBG (> 100 mg/dL) (Table 2).

### Effect of supplementation with milk protein on fasting insulin

**Fig. 2:**
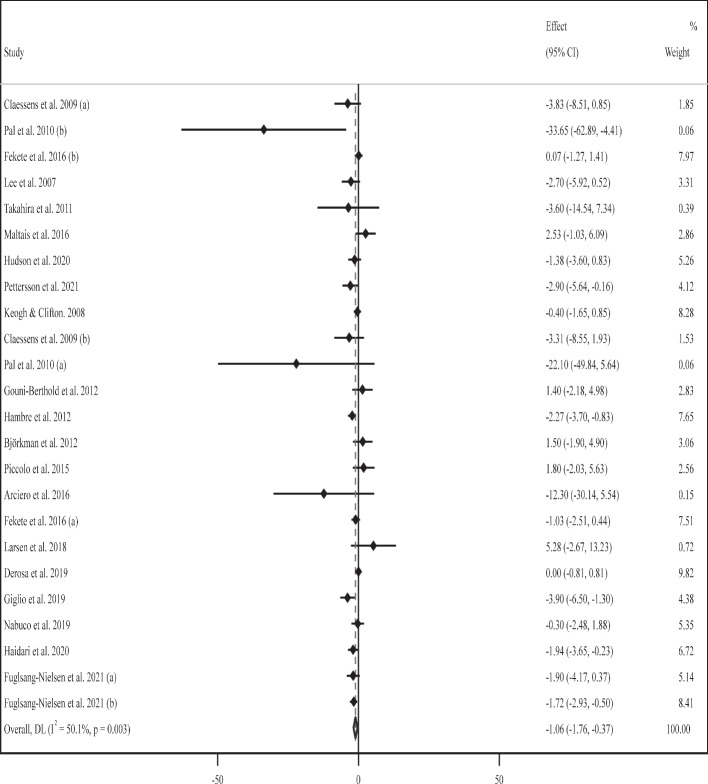
Forest plot for the effect of supplementation with milk protein on fasting insulin (uU/mL). Horizontal lines represent 95% confidence intervals (CIs). Diamonds represent pooled estimates from random-effects analysis. The effect column comprises weighted mean differences (WMDs) and 95% CIs

The effect of MP administration on serum fasting insulin values was evaluated in 20 RCTs [52–54, 57–59, 62, 64, 66, 67, 75–78, 80, 81, 83, 84, 86, 87] that involved 1100 participants (603 cases and 613 controls). A pooled analysis of 24 effect sizes indicated that the level of serum fasting insulin was considerably lower in the MP-treated group than in the control group (WMD: -1.06 uU/mL, 95% CI: -1.76, -0.36; *P* = 0.003). There was significant heterogeneity among RCTs (*I*^*2*^ = 50.1%, *P* = 0.003) (Fig. 2). Subgroup analyses depicted similar outcomes based on long-term supplementation with high or moderate daily doses of WP (≥ 60 or 30–60 g) among participants of both sexes (Table 2).

### Effect of supplementation with milk protein on serum HbA1c

**Fig. 3:**
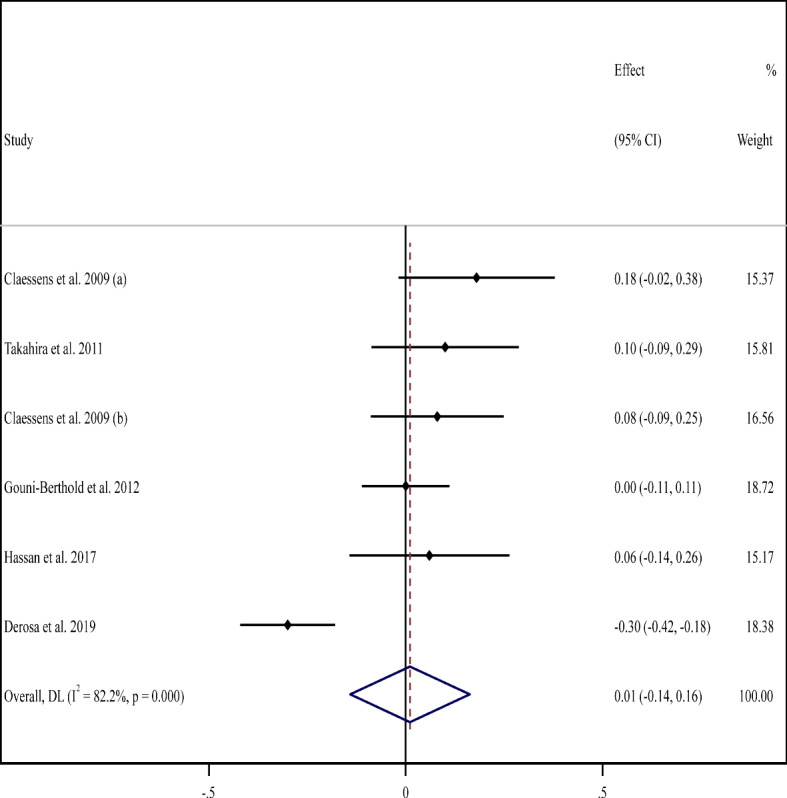
Forest plot for the effect of supplementation with milk protein on hemoglobin A1c (HbA1c) (%). Horizontal lines represent 95% confidence intervals (CIs). Diamonds represent pooled estimates from random-effects analysis. The effect column comprises weighted mean differences (WMDs) and 95% CIs

The meta-analysis of five studies (6 arms) [54, 57, 58, 70, 76] with 432 participants explored no significant changes in serum concentrations of HbA1c in the MP supplement-treated group compared to the untreated group (WMD: 0.01%, 95% CI: -0.14, 0.16; *P* = 0.89) with a high degree of heterogeneity between studies (*I*^*2*^ = 82%, *P* < 0.001) (Fig. 3). However, there was a substantial decline in serum levels of HbA1c among participants with normal baseline BMI based on sub-analyses (Table 2).

### Effect of supplementation with milk protein on HOMA-IR

**Fig. 4:**
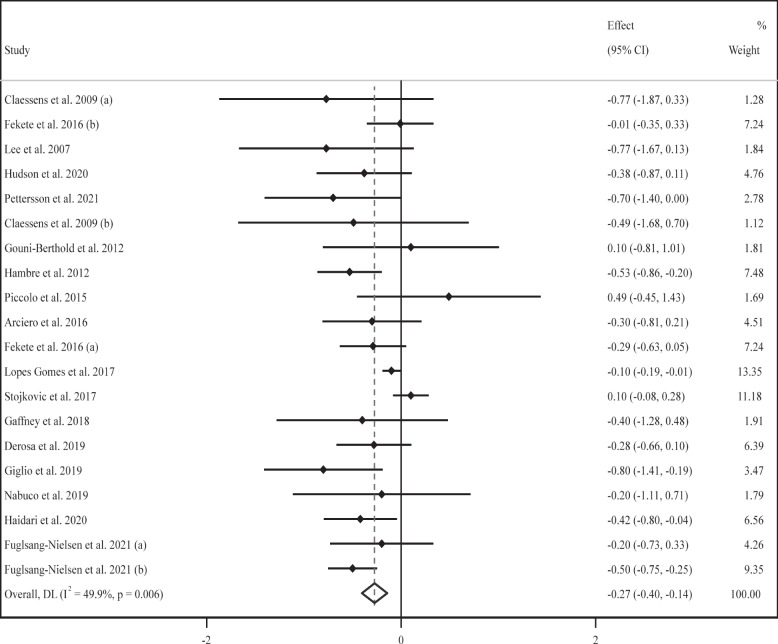
Forest plot for the effect of supplementation with milk protein on homeostasis model assessment of insulin resistance (HOMA-IR). Horizontal lines represent 95% confidence intervals (CIs). Diamonds represent pooled estimates from random-effects analysis. The effect column comprises weighted mean differences (WMDs) and 95% CIs

Seventeen trials [52, 54, 58, 59, 64, 66, 68, 69, 73, 76–78, 80, 81, 83, 84, 87] with 20 effect sizes and 940 participants revealed the impact of MP supplementation on HOMA-IR values. The meta-analysis displayed that the mean value of HOMA-IR was considerably lower in the experimental group than in the controls (WMD: -0.27, 95% CI: -0.40, -0.14; *P* < 0. 00.1) (Fig. 4). In addition, substantial heterogeneity was found between trials (*I*^*2*^ = 49.9%, *P* = 0.006). Subgroup analysis indicated that HOMA-IR values were significantly lower in the MP supplement-treated group than their untreated counterparts in short- and long-term supplementation (≤ 8 and > 8 weeks) with high or moderate doses (≥ 60 or 30–60 g/d) of MP or WP; similar outcomes were detected in subgroups including participants of both sexes with a normal or obese baseline BMI, and high or low baseline HOMA-IR values (> 2 or < 2) (Table 2).

### Publication bias

Visual inspection of the funnel plots displayed different degrees of asymmetry for all assessed outcomes (Supplemental Fig. 2). There was no publication bias for FBG, HbA1c, or fasting insulin outcomes based on Begg’s and Egger's tests. However, there was publication bias for the HOMA-IR outcome (*P* = 0.033) according to Egger's test.

### Linear and non-linear dose–response relations

There was no linear (Supplemental Figs. 5 and 6) or non-linear (Supplemental Figs. 3 and 4) relationship between changes in trial duration or doses of MP supplement and serum HbA1c values based on the dose–response assessment. There was a substantial non-linear association between changes in the duration of the intervention and serum levels of FBG (*P* = 0.005; Supplemental Fig. 4A) and fasting insulin (*P* = 0.03, Supplemental Fig. 4B), as well as between doses of MP supplements and changes in HOMA-IR values (*P* = 0.02; Supplemental Fig. 3D).

### Sensitivity analysis

Excluding any specific study did not affect the evaluated outcomes (values of fasting insulin, HOMA-IR, HbA1c, and FBG) based on sensitivity analysis.

## Discussion

This dose–response meta-analysis of 36 RCTs evaluated the impact of MP administration on the glycemic parameters in adults. It was indicated that supplementation with milk protein effectively reduced the levels of HOMA-IR, fasting insulin, and FBG while making no remarkable changes in serum HbA1c values. However, there was a significant decline in serum levels of HbA1c among participants with normal baseline BMI based on sub-analyses.

A subgroup analysis revealed that HOMA-IR values were significantly lower in the MP supplement-treated group than their untreated counterparts in short- and long-term supplementation (≤ 8 and > 8 weeks) with high or moderate doses (≥ 60 or 30–60 g/d) of MP or WP; similar outcomes were detected in subgroups among participants of both sexes with a normal or obese baseline BMI, and high or low baseline HOMA-IR values (> 2 or < 2). In addition, it explored that serum FBG levels were considerably reduced upon short-term administration (≤ 8 weeks) of a low daily dose of WP (< 30 g) among female participants with normal or overweight BMI, and higher baseline FBG (> 100 mg/dL). Furthermore, the levels of serum fasting insulin were remarkably decreased during long-term supplementation with high or moderate daily doses of WP among participants of both sexes. The dose–response assessment showed a significant non-linear relationship between changes in the intervention duration and serum concentrations of FBG and fasting insulin, as well as between doses of MP supplements and changes in HOMA-IR values.

This meta-analysis suggests that supplementation with MP could significantly ameliorate some glycemic parameters (fasting insulin, HOMA-IR, and FBG) of adults. However, the improvements were relatively small and might not be clinically significant. The minimum clinically important difference (MCID) for FBG and HA1C is between ≥ 14 and ≥ 0.5% mg/dL, respectively [88, 89]. The hypoglycemic effects of MP supplements are lower than MCID, which means that the impact is clinically insignificant.

A meta-analysis of 22 RCTs indicated that WP administration significantly decreased the values of HOMA-IR, HBA1c, and fasting insulin in patients with metabolic syndrome, but did not have any impact on FBG levels [90]. A systematic review of 58 RCTs explored that WP exerts a significant impact on glycemic control primarily by stimulating incretins and insulin secretion, suppressing appetite, and slowing down gastric emptying [39]. In addition, a comprehensive review of the literature stated the positive impacts of WP supplementation on improving postprandial glycemic control in the short term based on a few studies [91]. Another meta-analysis of five RCTs revealed that premeal WP supplementation is beneficial to ameliorate postprandial glycemia in patients with well-controlled or mild T2DM without significant adverse effects [37]. Some observational studies have reported a negative correlation between milk consumption and hyperglycemia [92, 93]. A prospective study displayed that a higher intake of dairy products was related to a lower 9-year incidence of hyperglycemia [92]. Another prospective cohort study among 15,512 adults in China (median follow-up of 9 years) declared that dairy consumption such as liquid milk and milk powder, was inversely associated with reduced diabetes risk [93]. However, most previous interventional trials failed to highlight the findings from observational studies regarding supplementation with MP.

It has been revealed that the insulinotropic impact of MP is related to certain AAs, in particular BCAAs [94]. Leucine induces glutamate dehydrogenase activity in β-cells that leads to an enhancement in Krebs cycle activity and insulin production [95]. In addition, WP as a fast digestible protein and a remarkable source of BCAAs promotes the circulation and release of insulin that may reduce postprandial hyperglycemia [90]. Bioactive peptides also induce the release of incretin hormones including glucagon‐like peptide‐1 (GLP‐1) and glucose-dependent insulinotropic polypeptide (GIP) that play a significant role in the enhancement of insulin resistance [96].

The maintenance of glucose levels involves a complex interaction between insulin-sensitive peripheral tissues and pancreatic β-cells [97]. The AAs are vital nutrients that may induce a diversity of indirect and direct impacts at the organismal and cellular levels [97]. However, there is a debate regarding the optimal amount of dietary protein for T2DM patients [97, 98]. It has been proposed that excessive amounts of AAs may reduce insulin-stimulated glucose uptake and increase insulin resistance [97]. A meta-analysis of eight RCTs explored that the consumption of proteins, particularly animal proteins, may be associated with an increased risk of T2DM [99].

The current study demonstrated a considerable reduction in serum FBG levels in RCTs that were short-term interventions with low-dose WP administration. Previous studies have revealed that the short-term effects of WP supplementation were equivalent to insulin therapy or sulfonylurea for the treatment of hyperglycemia in T2DM patients [28, 100, 101]. These promising results have only been displayed in short-term clinical trials. Therefore, short-term epidemiological and clinical evidence suggests that dairy proteins may ameliorate hyperglycemia. Although the outcomes of the present study proposed that the insulin-lowering effects of MP supplements are more efficient at higher doses and long-term interventions, further long-term RCTs are essential to confirm the proper efficacy, safety, and dosage of consistent consumption of MP supplements.

There were several strengths in the present systematic review and meta-analysis. This study is the first dose–response meta-analysis to evaluate the impact of supplementation with MP on the glycemic profile of adults. There was no restricted search period for selecting all eligible RCTs in a systematic search. In addition, a considerable number of studies were included in the analysis. Most of the RCTs in this meta-analysis had good or fair quality. Several limitations to the outcomes of this study should be considered. Dietary protein and carbohydrate intakes of participants were not reported in the majority of studies. The included RCTs in this meta-analysis had different control or non-intervened groups. Furthermore, there was considerable heterogeneity between trials related to each outcome. Therefore, a pre-defined subgroup analysis was employed to identify the cause of heterogeneity based on several variables, including supplement dose, intervention length, baseline BMI, baseline glycemic status, and gender of participants.

In conclusion, supplementation with MP may ameliorate the glycemic profile in adults by reducing the values of HOMA-IR, FBG, and fasting insulin. However, glycemic changes following MP administration were lower than MCID; therefore, its hypoglycemic effects were minor and may not reach clinical importance. Additional RCTs with longer durations are expected to confirm these findings.

### Supplementary Information

**Additional file 1:** **Supplemental Table 1.** Risk of bias assessment for included RCTs in the meta-analysis. **Supplemental Table 2.** GRADE assessment. **Supplemental Fig. 1.** Flow diagram of study selection. **Supplemental Fig. 2.** Funnel plots for the effect of supplementation with milk protein on (A) fasting blood glucose (FBG) (B) fasting insulin (C) hemoglobin A1c(HbA1c), and (D) homeostasis model assessment of insulin resistance (HOMA-IR). **Supplemental Fig. 3.** Non-linear dose-response association between dose (gr/day) of supplementation with milk protein and absolute mean differences in (A) fasting blood glucose (FBG) (B) fasting insulin (C) hemoglobin A1c(HbA1c), and (D) homeostasis model assessment of insulin resistance (HOMA-IR). The 95% CI (confidence interval) is demonstrated in the shaded parts. **Supplemental Fig. 4.** Non-linear association between duration of the supplementation with milk protein (weeks) and absolute mean differences in (A) fasting blood glucose (FBG) (B) fasting insulin (C) hemoglobin A1c(HbA1c), and (D) homeostasis model assessment of insulin resistance (HOMA-IR). The 95% CI (confidence interval) is depicted in the shaded parts. **Supplemental Fig. 5.** Linear dose-response association between dose (gr/day) of supplementation with milk protein and absolute mean differences in (A) fasting blood glucose (FBG) (B) fasting insulin (C) hemoglobin A1c(HbA1c), and (D) homeostasis model assessment of insulin resistance (HOMA-IR). **Supplemental Fig. 6.** Linear association between duration of the supplementation with milk protein (weeks) and absolute mean differences in (A) fasting blood glucose (FBG) (B) fasting insulin (C) hemoglobin A1c (HbA1c), and (D) homeostasis model assessment of insulin resistance (HOMA-IR).

## Data Availability

The datasets analyzed during the current study are presented in the manuscript.

## Abbreviations

RCT: Randomized controlled trial
FBG: Fasting blood glucose
HbA1c: Hemoglobin A1c
HOMA-IR: Homeostasis model assessment of insulin resistance
T2DM: Type 2 diabetes mellitus
HBG: High blood glucose
CVDs: Cardiovascular diseases
CHO: Carbohydrates
GI: Glycemic index
WP: Whey protein
CP: Casein Protein
MP: Milk Protein
PRISMA: Preferred reporting items for systematic reviews and meta-analyses
MeSH: Medical subject headings
RoB: Risk of bias
WMD: Weighted mean difference
SD: Standard deviation
CI: Confidence interval
ALS: Amyotrophic lateral sclerosis
GRADE: Grading of recommendations assessment, development, and evaluation
MCID: Minimum clinically important difference
BMI: Body mass index
PROSPPERO: International prospective register of systematic reviews
