# Supplementary material for: Effects of supplementation with milk protein on glycemic parameters: a GRADE-assessed systematic review and dose–response meta-analysis
Source: Nutr J. 2023 Oct 6;22:49. doi: 10.1186/s12937-023-00878-1 (PMC10557355; doi:10.1186/s12937-023-00878-1)
Supplement: Supplementary file 1 — Additional file 1: Supplemental Table 1. Risk of bias assessment for included RCTs in the meta-analysis. Supplemental Table 2. GRADE assessment. Supplemental Fig. 1. Flow diagram of study selection. Supplemental Fig. 2. Funnel plots for the effect of supplementation with milk protein on (A) fasting blood glucose (FBG) (B) fasting insulin (C) hemoglobin A1c(HbA1c), and (D) homeostasis model assessment of insulin resistance (HOMA-IR). Supplemental Fig. 3. Non-linear dose-response association between dose (gr/day) of supplementation with milk protein and absolute mean differences in (A) fasting blood glucose (FBG) (B) fasting insulin (C) hemoglobin A1c(HbA1c), and (D) homeostasis model assessment of insulin resistance (HOMA-IR). The 95% CI (confidence interval) is demonstrated in the shaded parts. Supplemental Fig. 4. Non-linear association between duration of the supplementation with milk protein (weeks) and absolute mean differences in (A) fasting blood glucose (FBG) (B) fasting insulin (C) hemoglobin A1c(HbA1c), and (D) homeostasis model assessment of insulin resistance (HOMA-IR). The 95% CI (confidence interval) is depicted in the shaded parts. Supplemental Fig. 5. Linear dose-response association between dose (gr/day) of supplementation with milk protein and absolute mean differences in (A) fasting blood glucose (FBG) (B) fasting insulin (C) hemoglobin A1c(HbA1c), and (D) homeostasis model assessment of insulin resistance (HOMA-IR). Supplemental Fig. 6. Linear association between duration of the supplementation with milk protein (weeks) and absolute mean differences in (A) fasting blood glucose (FBG) (B) fasting insulin (C) hemoglobin A1c (HbA1c), and (D) homeostasis model assessment of insulin resistance (HOMA-IR). [file 12937_2023_878_MOESM1_ESM.docx]

**Online Supporting Material**

**Supplemental Table 1** Risk of bias assessment for included RCTs in the meta-analysis on the impact of supplementation with milk protein on glycemic parameters

| **Author, year** | **Bias arising from the randomization process (Allocation bias)** | **Bias due to deviations from the intended interventions (Performance bias)** | **Bias due to missing outcome data (Attrition bias)** | **Bias in the measurement of the Outcome (Detection bias)** | **Bias in the selection of reported results (Reporting bias)** | **The overall risk of bias** |
| --- | --- | --- | --- | --- | --- | --- |
| Lee et al. 2007 | L | L | L | L | L | L |
| Keogh & Clifton. 2008 | L | L | U | L | L | U |
| Claessens et al. 2009 | H | L | L | L | L | H |
| Pal et al. 2010 | H | L | U | L | L | H |
| Silva et al. 2010 | L | L | L | L | L | L |
| Pal et al. 2010 | H | L | U | L | L | H |
| Takahira et al. 2011 | L | L | L | L | L | L |
| Gouni-Berthold et al. 2012 | L | L | L | L | L | L |
| Ahmadi Kani Golzar et al. 2012 | H | L | L | L | L | H |
| Hambre et al. 2012 | H | L | L | L | L | H |
| Sheikholeslami Vatani et al. 2012 | H | L | L | L | L | H |
| Björkman et al. 2012 | L | L | U | L | L | U |
| Rambousková et al. 2014 | H | L | L | L | L | H |
| Piccolo et al. 2015 | L | L | U | L | L | U |
| Tahavorgar et al. 2015 | L | L | L | L | L | L |
| Fekete et al. 2016 | L | L | L | L | L | L |
| Arciero et al. 2016 | H | L | U | L | L | H |
| Maltais et al. 2016 | L | L | L | L | L | L |
| Stojkovic et al. 2017 | L | L | L | L | L | L |
| Lockwood et al. 2017 | L | L | U | L | L | U |
| Ottestad et al. 2017 | L | L | L | L | L | L |
| Lopes Gomes et al. 2017 | H | L | L | L | L | H |
| Hassan et al. 2017 | H | L | L | L | L | H |
| Larsen et al. 2018 | H | L | U | L | L | H |
| Sharp et al. 2018 | L | L | L | L | L | L |
| Gaffney et al. 2018 | L | L | L | L | L | L |
| Nabuco et al. 2019 | L | L | L | L | L | L |
| Giglio et al. 2019 | L | L | L | L | L | L |
| Derosa et al. 2019 | L | L | L | L | L | L |
| Yang et al. 2019 | L | L | L | L | L | L |
| Hudson et al. 2020 | L | L | L | L | L | L |
| lefferts et al. 2020 | L | L | U | L | L | U |
| Haidari et al. 2020 | H | L | L | L | L | H |
| Fuglsang-Nielsen et al. 2021 | L | L | L | L | L | L |
| Pettersson et al. 2021 | H | L | L | L | L | H |
| Teixeira et al. 2022 | L | L | L | L | L | L |

*Abbreviations:* *L* low risk of bias, *H* high risk of bias, *U* unclear risk of bias (Some concerns)

**Supplemental Table 2** GRADE assessment

| Outcomes | Risk of bias | Inconsistency | Indirectness | Imprecision | Publication Bias | Quality  of evidence |
| --- | --- | --- | --- | --- | --- | --- |
| FBG | No serious limitation | Very serious limitation ^1^ | No serious limitation | No serious limitation | No serious limitation | ⊕⊕⊖⊖  Moderate |
| Fasting Insulin | No serious limitation | Serious limitation ^1^ | No serious limitation | No serious limitation | No serious limitation | ⊕⊕⊕⊖  High |
| HbA1c | No serious limitation | Very serious limitation ^1^ | No serious limitation | Serious limitation ^3^ | No serious limitation | ⊕⊖⊖⊖  Low |
| HOMA-IR | No serious limitation | Serious limitation ^1^ | No serious limitation | No serious limitation | Serious limitation ^4^ | ⊕⊕⊖⊖  Moderate |

1. There is high heterogeneity (I^2^>75%).
2. There is moderate heterogeneity (I^2^>40%).
3. There is no evidence of significant effects of milk protein supplementation.
4. There is a significant publication bias based on Egger’s test.

Identification

Records identified after databases search (n=15632)

PubMed (n=6683), ISI Web of Science (n=3462), and Scopus (n=5487)

Studies screened (n=10394)

Records excluded

Not reported desired data (n=71)

Articles were excluded after evaluating the title and abstract (n=10287)

Full-text article for eligibility (n=107)

Studies excluded after duplicates removal (n=5238)

Screening

Eligibility

Studies included in systematic review and meta-analysis

(n=36)

Included

**Supplemental Fig. 1** Flow diagram of study selection

**A)**

**B)**

**C)**

**D)**

**Supplemental Fig. 2** Funnel plots for the effect of supplementation with milk protein on (**A**) fasting blood glucose (FBG) (**B**) fasting insulin (**C**) hemoglobin A1c(HbA1c), and **(D)** homeostasis model assessment of insulin resistance (HOMA-IR)

**A)**

**B)**

**C)**

**D)**

**Supplemental Fig.3** Non-linear dose-response association between dose (gr/day) of supplementation with milk protein and absolute mean differences in (**A**) fasting blood glucose (FBG) (**B**) fasting insulin **(C)** hemoglobin A1c(HbA1c), and **(D)** homeostasis model assessment of insulin resistance (HOMA-IR). The 95% CI (confidence interval) is demonstrated in the shaded parts

**A)**

**B)**

**C)**

**D)**

**Supplemental Fig. 4** Non-linear association between duration of the supplementation with milk protein (weeks) and absolute mean differences in (**A**) fasting blood glucose (FBG) (**B**) fasting insulin **(C)** hemoglobin A1c(HbA1c), and **(D)** homeostasis model assessment of insulin resistance (HOMA-IR). The 95% CI (confidence interval) is depicted in the shaded parts

**A)**

**B)**

**C)**

**D)**

**Supplemental Fig. 5** Linear dose-response association between dose (gr/day) of supplementation with milk protein and absolute mean differences in (**A**) fasting blood glucose (FBG) (**B**) fasting insulin **(C)** hemoglobin A1c(HbA1c), and **(D)** homeostasis model assessment of insulin resistance (HOMA-IR)

**A)**

**B)**

**C)**

**D)**

**Supplemental Fig. 6** Linear association between duration of the supplementation with milk protein (weeks) and absolute mean differences in (**A**) fasting blood glucose (FBG) (**B**) fasting insulin **(C)** hemoglobin A1c (HbA1c), and **(D)** homeostasis model assessment of insulin resistance (HOMA-IR)
